# Cell Therapy Transplant Canada (CTTC) Consensus-Based Review for the Use of Supportive Care and Topical Therapies for Chronic Graft-Versus-Host Disease

**DOI:** 10.3390/curroncol33090548

**Published:** 2026-09-10

**Authors:** Kylie Lepic, Joseph Aziz, Gizelle Popradi, Christopher Lemieux, Jennifer White, Stephanie Maier, Mohamed Elemary, Kirk Schultz, Kristjan Paulson, Ram Vasudevan Nampoothiri, David Allan, Dennis Dong Hwan Kim

**Affiliations:** 1 Juravinski Cancer Centre, McMaster University, Hamilton, ON L8V 5C2, Canada; azizjo@hhsc.ca; 2 McGill University Health Centre, Montreal, QC H4P 2P5, Canada; 3 CHU de Québec, Université Laval, Québec, QC G1R 2J6, Canada; 4 Vancouver General Hospital, British Columbia Cancer Agency, University of British Columbia, Vancouver, BC V5Z 1M9, Canada; jennifer.white1@bccancer.bc.ca; 5 Cell Therapy Transplant Canada, Winnipeg, MB R3P 2R8, Canada; smaier@cttcanada.org; 6 Saskatchewan Cancer Agency, University of Saskatchewan, Saskatchewan, SK S7N 4H4, Canada; mohamed.elemary@saskcancer.ca; 7 British Columbia Children’s Hospital, University of British Columbia, Vancouver, BC V6H 3N1, Canada; kirk.schultz@ubc.ca; 8 Cancer Care Manitoba, University of Manitoba, Winnipeg, MB R3E 0V9, Canada; kpaulson@cancercare.mb.ca; 9 Ottawa Hospital Research Institute, University of Ottawa, Ottawa, ON K1H 8L6, Canada; rvasudevan@toh.ca (R.V.N.); daallan@toh.ca (D.A.); 10 Princess Margaret Cancer Centre, University Health Network, University of Toronto, Toronto, ON M5G 2M9, Canada; dr.dennis.kim@uhn.ca

**Keywords:** chronic GvHD, allogeneic cell transplantation, supportive care, topical, lifestyle modifications

## Abstract

This is a consensus-based Canadian review with the primary purpose of expanding knowledge regarding supportive and topical therapies for chronic graft vs. host disease (cGvHD). There is often a lack of confidence in treating certain organ specific manifestations, and access to specialists with organ specific expertise varies across the country. This paper covers information gained from a literature review and Canadian transplanter expertise and will serve as a guide for hematopoietic cell transplant healthcare providers to topical cGvHD therapies accessible in Canada. We will review common symptoms and signs focused specifically on skin, mouth, eye and genital tract cGvHD and on lifestyle modifications and topical treatments as an adjunct to our recent publication on systemic treatment for cGvHD.

## Simple Summary

Chronic graft-versus-host disease (cGvHD) is an important complication of allogeneic stem cell transplantation. It is a disease associated with significant morbidity, and as such there is a need to ensure healthcare providers are well equipped with information on how to treat patients holistically. Topical therapies and lifestyle modifications can play important roles in cGvHD care and are the focus of this manuscript.

## 1. Introduction

Chronic graft vs. host disease (cGvHD) is a complication of hematopoietic cell transplantation (HCT) that occurs when donor-derived immune cells interact with the cell populations and tissues of the recipient. This leads to inflammation, immune dysregulation of T and B cells, defective tissue repair, and ultimately results in fibrosis [1]. Chronic graft vs. host disease is estimated to occur in as many as 70% of allogeneic transplantations [2]. Fortunately, the incidence of cGvHD is decreasing with advances in prophylactic immunosuppression strategies; however, it remains a major cause of morbidity and mortality post-transplant. Also, the quality of life of patients with cGvHD is significantly lower with increasing severity of cGvHD [3,4,5,6].

Treatment of GvHD is guided by the severity of disease as determined by NIH scoring [3,7,8]. The CTTC has recently published guidelines on systemic treatment [9]. The Cell Therapy Transplant Canada (CTTC) Consensus-Based Guideline 2024 for the Management and Treatment of Chronic Graft-Versus-Host Disease and Future Directions for Development provides a comprehensive review of the biology, diagnosis, scoring and grading, systemic treatment strategies and monitoring of long-term sequelae. The NIH 2014 Ancillary Therapy and Supportive Care Working Group Report provides a thorough and extensive background on the topic of supportive therapies. In this paper, we will focus on a practical reference for topical treatments and lifestyle interventions for four of the most common manifestations: skin, eye, oral and genital tract.

Patients with moderate-to-severe-grade cGvHD require systemic therapy. Patients with mild-grade disease but who have high-risk features also require systemic therapy. For patients with low-global-severity scores, it is preferable provide treatment with topical therapies alone to avoid the side effects of systemic immunosuppression. However, it is important to note that all patients with cGvHD, even those already on systemic treatment, can benefit from ancillary topical and supportive therapies.

Patients with cGvHD are best served by a treatment approach involving integrated care from multidisciplinary teams including transplant teams as well as other experts (i.e., Dermatology, Ophthalmology, Oral Medicine, Gynecology/Urology, Gastroenterology, Hepatology, Nephrology, Neurology, etc.). Additionally, allied health professionals including physiotherapists, dieticians, massage therapists, psychologists, pharmacists and social workers have important roles in the overall care of patients with cGvHD.

## 2. Methods

This manuscript is a project of “Cell Therapy Transplant Canada’s Guideline Development Committee”. The purpose of this Committee is to provide a platform for knowledge sharing, collaboration, education and standards for the best practice for CTTC membership and the field of CTT. Members of the Working Committee included transplant physicians who diagnose and treat cGvHD in their practices. The terms of reference for the Committee included that it must contain a minimum of 3 representatives from different Canadian CTT centres contributing to the project. For this manuscript, 9 different centres participated. Of note, we were not able to engage any subspecialists which highlights the rarity of expertise in the treatment of cGvHD. The first draft was established at Hamilton Health Sciences. Contributing members from the CTTC Guideline DevelopmentSl Committee all shared their feedback and centre expertise. Consensus was obtained by circulation and non-objection. The Guideline Development Committee members have read, reviewed, and approved the final draft. We searched Pubmed from 1990 to 2026 to find English language articles with search terms including “cGvHD”, “topical and supportive care for cGvHD”, “eye cGvHD”, “mouth cGvHD”, “skin cGvHD”, and “genital tract cGvHD”.

## 3. Skin cGvHD

Chronic skin manifestations of GvHD include various sclerotic and non-sclerotic phenotypes. Lichen planus-like eruptions and poikiloderma are the most common non-sclerotic manifestations in addition to psoriasis-like, eczema-like, and keratosis pilaris-like lesions, severe dryness, morphea, hyperpigmentation or hypopigmentation, telangiectasia, erythema and pruritus. Sclerodermatous changes are characterized by fibrosis which may be superficial and localized or deeply sclerotic. It is important to note that deep sclerosis and fasciitis require systemic therapy as a mainstay of treatment. Nail changes such as dystrophy or changes in nail texture or shape and alopecia can also be seen.

### 3.1. Lifestyle Modifications and Supportive Care for Chronic Skin GvHD

Sun exposure can be a trigger or can potentially exacerbate GvHD in addition to increasing the risk of skin cancer, a common secondary malignancy seen post-transplant [10,11]. Basal cell carcinoma is the most common skin cancer observed post-transplant followed by squamous cell carcinoma and melanoma [11]. It is important that patients are educated regarding sun-protective measures including sun avoidance, protective clothing and the appropriate use of sunscreens that protect against both ultraviolet A (UVA) and ultraviolet B (UVB) radiation. Vitamin D supplementation should be considered.

Chronic GvHD can lead to brittle nail texture; thus, nail hardeners/strengtheners can create a barrier to prevent tearing. Moisturizers and emollients can restore the skin barrier, alleviating dryness and irritation associated with cGvHD. Fragrance-free or formulations designed for sensitive skin are also important. Avoidance of restrictive clothing at affected sites is recommended. Stretching exercises, massage or physical therapy may help loosen sclerosis and improve range of motion. Strength and movement exercises such as yoga, Tai Chi or Qi Gong have the potential to improve flexibility, strength and balance in those with sclerosis. These adjunctive exercises also have the benefit of muscle strengthening for those with muscle weakness resulting from previous systemic steroid exposure.

### 3.2. Local Treatments for Chronic Skin GvHD

Topical steroids are considered the first-line topical therapy for skin cGvHD based on consensus. The formulation and dose depend on location on the body (Table 1). For areas such as the trunk and extremities, higher doses can be started for 2 weeks and then tapered to lower doses once a response has been achieved. Lower-potency topical corticosteroids such as topical hydrocortisone 1.0% are preferred for long-term use on the face, axillae, and groin. Prolonged use of topical steroids can lead to skin atrophy and telangiectasia.

Topical calcineurin inhibitors, including tacrolimus ointment 0.1% and topical pimecrolimus, have also demonstrated efficacy in topical treatment and can be used in areas where steroid side effects are undesired (i.e., face, lips) or as alternatives to topical steroids as they do not carry the aforementioned side effects [12,13,14]. However, cautious use of topical tacrolimus on large surface areas should be exercised as systemic absorption can result in toxic levels in the blood [15].

Newer potential treatment options for skin GvHD currently under investigation include topical ruxolitinib. In a single-centre trial reported in abstract form, 24 patients applied ruxolitinib 1.5% cream to one side of the face or body and vehicle cream to the other; the treated side showed greater reduction in affected BSA at day 28 [16].

Phototherapy can cause apoptosis of pathogenic T cells and the expansion of regulatory T cells and as such it can be used for cGvHD of the skin; however, it requires the care of Dermatology teams and can be difficult to access offices that have the appropriate equipment. For chronic cutaneous sclerotic GvHD, it is recommended to use the deeper penetrating UVA with a wavelength of 340–400 nm [17]. For chronic cutaneous non-sclerotic or superficially sclerotic cGvHD, it is recommended to use narrowband UVB 311–313 nm [17]. Of note, there is potential concern for increased risk of skin malignancies with phototherapy.

Pruritus with GvHD can significantly impact quality of life. Treatment options include ensuring appropriate levels of hydration with emollients, topical steroids, and narrowband UVB phototherapy as well as antihistamines and gabapentinoids. Aprepitant has been used for chronic pruritus arising from various systemic conditions but there is no evidence for its use in cGvHD. For non-cicatricial alopecia (such as androgenetic alopecia or telogen effluvium), minoxidil 1.25–5 mg daily can be helpful. This is an off-label use of the product and side effects include fluid retention, hypertrichosis, reflex tachycardia and pericardial effusion.

Post-inflammatory hyperpigmentation (PIH) can be challenging to manage, and generally resolves spontaneously over months to years. If patients wish to treat PIH, short-term use (i.e., 8 weeks to avoid ochronosis) of topical therapies including retinoids (such as tretinoin, adapalene, or tazarotene) or steroids can be considered. An example of such a formulation includes hydroquinone 4%, tretinoin 0.05%, and fluocinolone 0.01% [17].

### 3.3. Additional Considerations for Chronic Skin GvHD

A potential consequence of sclerodermatous change can be skin ulceration. It is important to monitor this in case of soft tissue infection. Patients should be aware of other skin complications of transplant including the risk of secondary skin cancers, and suspicious lesions should be biopsied. Thus, close monitoring of other long-term sequelae from skin GvHD is important.

## 4. Eye GvHD

Chronic GvHD of the eye can affect multiple parts of the eye including the conjunctiva, cornea, eyelid, meibomian gland, nasolacrimal duct and lacrimal glands. There are several mechanisms behind ocular GvHD. Donor-derived T cells infiltrate the lacrimal glands and activation of the renin–angiotensin–aldosterone system contributes to lacrimal gland fibrosis impacting tear production. Chronic blepharitis damages the meibomian glands decreasing the quality and quantity of meibum and resulting in increased evaporation. Inflammation can also cause epithelial sloughing and fibrosis which can ultimately result in scarring and anatomic changes. With respect to the cornea, immune-mediated damage as well as a reduction in corneal nerve density can lead to filamentary keratopathy, superior limbic keratoconjunctivitis, and recurrent epithelial defects, and in severe cases, corneal ulcers and perforation [18,19]. Symptoms can manifest as dry, gritty, or painful eyes but can result in discomfort, impaired vision, and reduced quality of life. Of note, most patients with advanced ocular manifestations usually require systemic therapy in addition to topical care.

### 4.1. Lifestyle Modifications and Supportive Care for Eye GvHD

Protective eyewear such as moisture-chamber or wraparound glasses can decrease evaporation from wind and sunglasses can help photophobia. Avoidance of low-humidity environments may be beneficial.

Nutritional supplements such as fish oil (omega 3 fatty acids) or flaxseed oil (2000 mg/d) may be beneficial due to their anti-inflammatory properties [19].

It is important to ensure appropriate lid hygiene to prevent lid margin infection. Warm compresses, lid scrubs or sprays and gentle massage can help to prevent meibomian gland dysfunction, enhance tear film quality and reduce tear evaporation.

### 4.2. Treatments for Eye GvHD

Preservative-free artificial tears can provide moisture and reduce friction and irritation on the ocular surface, helping to alleviate symptoms. They should be used frequently throughout the day. Gels or ointments can be useful at night. Preservative-free formulations are preferred to avoid chemical irritation. Cyclosporine drops in doses of 0.05% have been reported to relieve dry eye symptoms and improve corneal fluorescein staining [20]. Topical tacrolimus 0.03% is also effective in controlling ocular surface inflammation in patients intolerant to cyclosporine (i.e., in case of burning sensation, redness, conjunctival lid swelling and itching) [21,22].

Topical steroid use is controversial as it is often associated with side effects such as increased intra-ocular pressure, cataracts, glaucoma, and infectious keratitis if used for longer durations. It has been shown to have beneficial effects in patients with cicatricial conjunctivitis associated with ocular GvHD whereas other studies have reported that topical steroids have minimal effect in ocular GvHD [23,24].

One single-centre sponsor-initiated study of 33 patients (22 in progesterone arm, 11 in placebo arm) demonstrated that twice-daily application of 1% progesterone gel to the lateral forehead significantly improved ocular signs and symptoms within 10 weeks [25].

Oral doxycycline can be used to treat meibomian gland dysfunction or associated rosacea. Targeted steroid ointment application to lid margins has also been very effective in treating symptoms of blepharitis [26]. Antibiotic ointment or eye drops can also be used for bacterial lid margin superinfection [27]. Medications such as cevimeline or pilocarpine can stimulate aqueous tear flow.

Both reversible punctal occlusion with silicone plug and irreversible thermal cauterization of the punctum can reduce tear drainage in patients with keratoconjunctivitis sicca [28,29].

Autologous eye drops which are prepared from a patient’s own serum and contain growth factors and nutrients that promote healing and lubrication can be used at dilutions ranging from 20 to 100% but access can be an issue. Autologous drops also require refrigeration which can be less convenient for some [30,31].

Scleral contact lenses provide a gas-permeable covering on the ocular surface to protect against mechanical injuries and tear evaporation, thus mitigating ocular symptoms, promoting the regeneration of the cornea and conjunctiva, and improving the quality of life of patients. However, access and cost can be prohibitive.

### 4.3. Additional Considerations for Eye GvHD

Cataracts can be observed in patients with cGvHD due to the use of systemic steroids and infectious keratitis may occur and should be considered on the differential if worsening of symptoms occurs.

## 5. Mouth GvHD

Signs of cGvHD of the oral cavity include xerostomia, mucosal inflammation, lichenoid lesions, and oral ulcers. Decreased oral opening secondary to sclerosis of oral and perioral tissues can also occur. cGvHD can impair oral intake and compromise nutrition due to dryness as well as pain and sensitivity.

### 5.1. Lifestyle Modifications and Supportive Care for Mouth GvHD

It is important to continue to maintain good dental hygiene as chronic dry mouth can increase risk of tooth decay and cavities. Oral hygiene practices tailored for sensitivity including use of a soft-bristle toothbrush, and non-mint, sodium lauryl sulfate-free and non-alcohol-based toothpastes are encouraged [32].

For xerostomia, lubricating rinses or sprays such as Biotene can help as can salivary stimulants containing xylitol such as sugar-free gum or lozenges. Lemon-flavoured or tart candies can also potentially stimulate saliva flow.

### 5.2. Treatment for Mouth GvHD

For lichenoid changes, the recommended treatment is a steroid rinse such as with dexamethasone 0.01% or clobetasol 0.05% oral suspension. It is important to advise patients to rinse and hold 5–10 mL in the mouth for 3–5 min and spit it out, repeating one to four times daily. Rinses should be followed by a 20 to 30 min period of no food or fluid intake. Note that oral candidiasis can be a complication, and an antifungal rinse post steroid rinse can be considered to help prevent this when prolonged use is required. Patients should also be aware that some systemic absorption of the steroid can occur. Topical calcineurin inhibitor rinses such as cyclosporine 100 mg/mL (10% *w*/*v*) 5 mL swished and expectorated three times daily can be used [33].

For ulceration, topical steroid gels such as clobetasol propionate gel 0.05% or tacrolimus gel 0.1% held in place with a gauze for 10–15 min can be used [32]. Night-time application of the gel to maximize medication exposure to the lesion (e.g., no displacement due to eating or drinking) is recommended. Intra-lesional steroid injections or autologous blood product patches have been used in practice but are not easily accessible.

For xerostomia, a trial of sialagogues such as pilocarpine or cevimeline for several weeks can be attempted and continued if beneficial. These agents generally work if saliva-producing cells remain in the salivary glands.

### 5.3. Additional Considerations for Mouth GvHD

It is important in cases of non-resolving oral lesions to consider the possibility of a secondary oral malignancy and send atypical lesions for assessment and biopsy. Regular visits to a dentist for routine care and oral cancer screening is recommended.

## 6. Genital Tract GvHD

Chronic GvHD of the genital tract is one of the most underreported forms of cGvHD. Signs and symptoms in female patients include irritation and erosion of the mucosa, fissures, leukokeratosis, labial or clitoral fusion, fibrous vaginal ring, vaginal shortening, vaginal adhesions and complete vaginal stenosis [34]. Patients may experience symptoms that include dryness, burning, pruritus, pain, dysuria, dyspareunia and sexual dysfunction. Chronic GvHD of the genital tract in males can present as inflammatory balanoposthitis, lichen sclerosus-like lesions, and phimosis. Erectile dysfunction is reported although this can be multifactorial in its etiology [35].

### 6.1. Lifestyle Modifications and Supportive Care for Genital Tract GvHD

Lifestyle considerations to prevent symptoms may include use of water-based lubricants during sexual activity and routine use of a dilator to prevent stenosis along with pelvic floor physiotherapy. Emollients like petrolatum, glycerin, and coconut oil may be applied to the external genitalia to provide relief from itching or irritation.

### 6.2. Treatment for Genital Tract GvHD

For GvHD of the vulva, direct topical application of high-potency steroids such as clobetasol propionate 0.05% to the lesions twice a day can be used. Hormonal therapy such as the use of topical estriol in the form of 1 mg cream or 1 mg suppositories on alternate days can prevent atrophy and maintain lubrication and vaginal elasticity [36].

For penile lesions, topical steroids can also be used. For lichenoid changes, topical calcineurin inhibitors can be applied to the penis as a second-line therapy.

In severe cases of strictures and adhesions, surgical intervention such as circumcision or lysis of adhesions may be required.

### 6.3. Additional Considerations for Genital Tract GvHD

Scarring can make the cervix inaccessible and impair the ability to complete cervical cancer screening (Table 2).

## 7. Conclusions

Chronic GvHD is a major cause of morbidity and associated with decreased quality of life post allogeneic transplant. Many of the symptoms are insidious and multi-organ dysfunction is common. Local/topical therapies play an important role in symptom management. A diagnosis of cGvHD in one organ should prompt detailed assessment of other organs. Patients should be monitored for GvHD at the transplant centre but in addition, routine organ-specific assessments by optometrists/ophthalmologists, dermatologists, urologists and gynecologists have an important role. Providing education regarding the signs and symptoms of cGvHD to patients and their caregivers will empower patients to connect with their care team when symptoms arise. In addition to cGvHD, routine screening can also pick up other long-term complications including secondary malignancies.

A limitation of this review is that it does not address detailed screening or other important supportive care measures such as infection prophylaxis, bone health, nutrition, pulmonary and psychosocial care. Also, despite efforts to engage subspecialty experts, the working group ultimately comprised transplant physicians alone. This reflects the limited availability of subspecialists with dedicated cGvHD experience in Canada, which is itself part of the rationale for this review, and readers should interpret the organ-specific recommendations as the considered view of transplant clinicians rather than of the relevant subspecialists.

Lifestyle modifications and local/topical therapies are integral parts of localized disease management and responses in cGvHD and can help reduce the need for systemic treatment and its associated side effects in transplant patients.

## Figures and Tables

**Table 1:** Topical steroid potency chart.

Potency	Product
Very High	Betamethasone dipropionate 0.05% Clobetasol propionate 0.05%
High	Fluocinonide 0.05% Mometasone furoate 0.1%
Moderate	Triamcinolone acetonide 0.1% Betamethasone valerate 0.1%
Low	Hydrocortisone 1% Desonide 0.05%

Potency class varies with vehicle, and ointments are more potent than creams or lotions.

**Table 2:** Common symptoms and summary of supportive treatments.

Symptom or Manifestation	Treatment	Reference(s)
Nail changes	Nail hardeners or polishes	
Alopecia	Minoxidil 1.25–5 mg po daily	
Dry eyes	Lid hygiene Warm compresses Omega-3 fatty acids or flaxseed oil Protective eyewear Viscous ointment Preservative-free artificial tears Cyclosporine eye drops 0.05% Topical tacrolimus drops 0.03% Progesterone gel 1% (applied to lateral forehead) Doxycycline Sialagogues (cevimeline or pilocarpine) Surgical–punctal plugs, debridement of filamentary keratitis Autologous serum eye drops Scleral lens prosthesis	[19,20,21,22,23,24,25,26,27,28,29,30,31]
Sclerotic skin changes/joint contracture	Moisturization Massage therapy Exercise program focusing on balance and movement Physiotherapy Topical steroids Topical calcineurin inhibitors	[12,13,14,17]
Post-inflammatory hyperpigmentation	Topical retinoids Topical steroids	[17]
Xerostomia	Xylitol gum Lemon candies Alcohol-free, sugar-free mouth washes (Biotene) Sialagogues (cevimeline or pilocarpine)	
Lichenoid oral changes	Steroid rinses Topical calcineurin inhibitors rinses	[32,33]
Oral ulcers	Topical steroid gel Topical calcineurin inhibitors gels Intra-lesional steroid injection Auto blood product patch Topical anesthetics	[32,33]
Pruritus	Topical steroids Antihistamines Gabapentinoids NBUVB phototherapy	
Vaginal dryness/irritation/narrowing	Water-based lubricants Emollients Routine dilatation Topical steroids Hormonal creams or suppositories	[36]
Penile lesions	Topical steroids	

## Data Availability

No new data were created or analyzed in this study. Data sharing is not applicable to this article.
